# The Present and Future of State Boards

**Published:** 1900-09

**Authors:** 


### Editorial.

### THE PRESENT AND FUTURE OF STATE BOARDS.

IT may seem to some impolitic to reopen a controversy with the organizations known by the name of State Boards of Dental Examiners, and this certainly would be unwise had there ever been a permanent peace established between the colleges and these political overseeing bodies. This, however, has never been the case; the quiet of the hour is that false quiet that precedes a more violent volcanic eruption.

The Boards have all had their usual spring examinations, and a certain per cent. of applicants have been rejected after having passed the various examinations of the college faculties.

To the average laymen this means a wholesome protection to the public and a renewed assurance of the value of this supervisory care in preventing unqualified men from entering the professional ranks. To the educators behind the examined students, it means simply that these students have succeeded or failed to answer a sufficient number of ten or a dozen questions in each branch. The success does not secure the dear public from the imposition of inefficiency, nor does it prove that the rejected are incapable of rendering good practical service.

The reason for this is obvious to the educator, but may not be equally clear to the man who looks solely upon the surface of things. The list of ten questions before alluded to may cover each of the subjects of anatomy, physiology, chemistry, materia medica, pathology, therapeutics, bacteriology, metallurgy, histology, oral surgery, mechanical and operative dentistry. It is equally clear that if a man should fail before the State board on anatomy and physiology, materia medica, or chemistry, it would so reduce his average that he would be rejected, although his standing in the other branches, especially the practical, might place him in the front rank in those qualifications that would enable him to render good service to suffering humanity. This does not seem to be considered by the examiners in their final decisions, and the young man is cast out, having spent his three years and earned his diploma in vain. The responsibility of thus wrecking a life may be appreciated by some of the boards of examiners, but it is feared there is a certain degree of satisfaction felt by others that they can thus show their great superiority to the colleges that trained these young men.

Superiority is a word of indefinite meaning. If, in their case, it is understood as a legal power given by the State Legislatures to do as they please, and ride rough-shod over the work of the colleges, then there will be no one to dispute the claim; but if it means that the average board is made up of elements of superior ability to the faculties of colleges, then there must be a decided dissent. Were it not that college men feel the seriousness of this whole business of re-examinations, the matter would be a subject for amusement.

The questions usually asked bear only a remote relation to the several subjects as practically taught. This could not be avoided when the source of most of these is understood. Quiz compends and ancient first editions of Harris's "Principles and Practice" are not exactly the best mines out of which to dig modern thoughts and modern science. It is not to be understood that this is applicable to all members of examining boards. Many of these are known to be cultured men, anxious to do the right thing in the right way. These, however, are handicapped by their lack of knowledge of what a modern dental college education means. They, as a rule, have never held close relation with professional pedagogical work, and therefore are, and have always been, out of touch with that knowledge which can only be received through intimate relations with student life and students' work. The questions given in these re-examinations lack life. They are apt to be general in character, striking, as it were, at the periphery of a subject rather than seeking the innermost central idea. The untrained examiner invariably seeks a question that when answered means nothing of real value, and which fails to determine the knowledge of the individual, but it gives a certain degree of satisfaction to the examiner, as it magnifies his self-consciousness of power.

The question of greater importance to the public, were it properly appreciated, would be, Do these re-examinations protect the people? As the boards are at present constituted, the answer must be in the negative. They not only do not protect, but experience has demonstrated that they are a menace to the very people they are supposed to serve.

It is difficult in the very best conducted schools, whether of dentistry or medicine, to prevent the man who will make a failure in practice from graduating. It is within the experience of every teacher that the brilliant mind will grasp all the subjects taught readily, but will fail in manipulative dexterity. On final examinations such a man will carry all before him in branches that require pure mental effort, while in the practical his work will fall below the average. What is to prevent such a man graduating either through a faculty or a board of examiners? This, however, is a trifling matter, for such a man, while not capable of doing much good, especially in dentistry, will not probably do much harm, for the public will soon take his measure and avoid him. The other man, with mind sluggish and perhaps untrained to study, will find it impossible to meet the requirements of the faculty, and should he pass this difficulty, will suffer wreck in the re-examination, and yet he may be brilliant in those things that serve the public best.

Before the boards were instituted dentistry flourished, and the public decided for itself that which was good or bad. There was not then a far-off cry of this public demanding that it should be protected. The cry came from within the ranks. It was not the people, but the dental profession, that needed protection. Somebody must be given power to stop the flood of dentists pouring over the country. What was the result? With great difficulty legislatures were influenced to pass laws regulating dentistry. Governors were empowered to appoint boards of examiners. Men were selected, good, bad, and indifferent, and the great reform began. It was prophesied that soon the quack would be run to earth, soon dental colleges would be forced to do as the boards might suggest, or go to the wall. Soon the number of graduates would be lessened, and soon the standard of the dental profession would be raised.

A quarter of a century has passed since the influence of State boards began to be seriously felt, and this constitutes a sufficient period to measure results. Twenty-five years ago there were a few cases in all large cities where men sought to secure practice by unethical methods, but these were so few, and, while annoying from a professional stand-point, they occasioned no marked deleterious influence. The result now, after this long period of law, is that the "Dental Parlor" is duplicated and reduplicated in every large city, and even in smaller towns where it previously had no existence. It may be said that this has no direct bearing on the laws; that this would have been the result had no laws governing dentistry been placed upon the statute-books. This is possibly true, but the laws have been a complete failure in the suppression of these, even in the one State that fully enforces its law. The graduates, instead of being fewer in number, have been enormously increased until even educators have become anxious as to the final result. To meet this and to make more thorough men, the standard of entrance has been raised in all the colleges of the country. The curriculum has been advanced and the time lengthened until it would seem impossible to go farther in this direction, and yet a still further increase in years is now demanded. For all these changes the boards take to themselves special credit. It is safe to assert that they had nothing to do with any advance. This came from within, and not from without, and would have been found necessary had the boards never been established. This is the status at present. The students are made to suffer, and the dental profession has gained nothing through this so-called supervisory power.

The fact that the State laws governing dentistry are not enforced to any appreciable extent is patent to every observer. It is possible, in some of the States possessing stringent laws, for a man to practise in spite of the boards, and this has frequently been done. The answer the boards make to this is, "We are not appointed for police work, and fulfil our duty when we have examined the students." This is true, and the enforcement of the law then devolves on others. The State officials whose duty it is take no interest in it, and will do nothing unless cases are brought directly to their notice, with proper evidence sufficient to secure conviction. The State societies have appointed committees, but they have failed to produce satisfactory results. The duty is disagreeable, and means large expenditure of money and time, and a certain loss of reputation. The result is that the law is not enforced. It would be perfectly easy in the State in which the writer lives for a man to practise dentistry without even a college training, and some have boldly declared they would practise in spite of the decision of the board. This is unquestionably a bad state of things, but it is not only the case, but there seems no prospect of any improvement. Better no law than its constant violation. New York is probably the only State where it would be dangerous to practise without authority. This is mainly due to one man. When he retires, it may not be possible to find another equally willing to assume the thankless task.

The future of these boards is a problem that will require the combined wisdom of all thinking minds. It is too late to expect these to be dispensed with. The laws will remain, and we must continue to have these bodies to deal with for many years to come. The question that interests us now is, Can there be any change made in the selection of those who will constitute these boards in the future?

It is no new thought that the men appointed on boards of examiners should themselves be forced to undergo an examination. To effect this has been deemed an impossibility, but it has never seemed to the writer a difficult problem. That the men to examine should themselves be examined requires no argument. The present method of selecting members of these boards is defective, even where the greatest care is taken by State societies, but those made exclusively by governors do not deserve a moment's consideration. They are intrinsically worthless, no matter how individually constituted. There seems to be but one way out of this difficulty, and that is to have all the laws so amended that State societies shall have the selection of a certain number, and from this list the governor to make selection. This is the present method adopted in several of the States. The writer would suggest a still further advance. Let the State societies amend the code of rules, so as to require all candidates for the State board of examiners to undergo an examination before being appointed. To meet this an examination board should be provided, to consist of a certain number of men taken from the college or colleges in the State where these exist, to make the necessary examinations into the fitness of the candidates for the work required. This would demand no change in existing laws. It will be said, You would never secure a board under such a rule. This would be true of the older men. It is well understood that they could not pass the simplest examination in the subjects previously named; indeed, their own questions would be a stumbling-block outside of their own specially prepared subject. There is, however, a large number of young men fresh from the more modern teaching of the schools who could and would submit themselves to such a board. There would be honor, as well as pecuniary inducement, for these younger men to make the effort. If such a plan succeeded, it would be a great advance over anything we have at present, although it is not contended that even this would make an ideal board. The board should then be supplemented by a salaried official whose duty it should be to hunt up cases and the evidence, and this man should not be a dentist in practice.

Something of the kind outlined here will have to be done if the future of the boards is to be anything more than a byword and reproach. At present they mean nothing but a worry, and oftentimes an injustice to the student and a continued aggravation to the teacher. The present arrangement is a crude effort that will not and ought not to stand the test of time, and the earlier the intelligent men connected with dental work and its progress take this seriously in hand, the better it will be for the future of the dental profession, in which all are interested and desirous of serving, that in the end it may advance beyond the criticism of the most exacting.

